# Hip Hemiarthroplasty: The Misnomer of a Narrow Femoral Canal and the Cost Implications

**DOI:** 10.7759/cureus.18971

**Published:** 2021-10-22

**Authors:** Sadhin Subhash, Maheswaran W Archunan, Nameer Choudhry, Justin Leong, Khaldoun Bitar, Sheryl Beh, Sarmila Tharmakulasingam, Sayam Subhash, David Melling, Ignatius Liew

**Affiliations:** 1 Trauma and Orthopaedics, Norfolk and Norwich University Hospital, Norwich, GBR; 2 Trauma and Orthopaedics, Whiston Hospital, St Helens and Knowsley Teaching Hospitals, Liverpool, GBR; 3 Trauma and Orthopaedics, Aintree University Hospital, Liverpool, GBR; 4 Cardiovascular, Duke-National University of Singapore (NUS), Singapore, SGP; 5 Oncology, Norfolk and Norwich University Hospital, Norwich, GBR; 6 Physiology, Government Medical College, Kollam, IND; 7 Orthopaedics, Addenbrooke's Hospital, Cambridge, GBR

**Keywords:** random allocation, research design, hemiarthroplasty, femur head, cost savings, template, outcome, hip fracture

## Abstract

Objective

Hemiarthroplasty has been identified as the treatment of choice for displaced intracapsular femoral neck fractures. A modular prosthesis is sometimes preferred for its sizing options in narrow femoral canals, despite its higher cost and no advantage in clinical outcomes. Thus, in this study, we investigated the factors affecting surgeons’ choice of prosthesis, hypothesizing that modular hemiarthroplasty is overused for narrow femoral canals compared to monoblock hip hemiarthroplasty.

Methods

A retrospective study of a regional level 1 trauma center was conducted. Patients who had sustained femoral neck fractures from March 2013 to December 2016 were included in this study. Inclusion criterion was modular hemiarthroplasty for a narrow femoral canal. A matched group of patients who underwent monobloc hemiarthroplasty (MH) was created through randomization. The main outcome measurements were sex, age, Dorr classification, and femoral head size. We measured the protrusion of the greater trochanter beyond the level of the lateral femoral cortex postoperatively. Modular hemiarthroplasty patients were templated on radiographs using TraumaCad for Stryker Exeter Trauma Stem (ETS®).

Results

In total, 533 hemiarthroplasty procedures were performed, of which 27 were modular for a narrow femoral canal. The ratio of modular to monobloc was 1:18. Average head size was 46.7 mm ± 3.6 mm for monobloc and 44.07 ± 1.5 for modular (P= 0.001). There were four malaligned stems in the monobloc group versus 14 in the modular group (P= 0.008). Unsatisfactory lateralization was noted in 18 patients (7 mm ± 2.9 mm) in the modular group compared with 8 (4.7 mm ± 3.9 mm) in the monobloc group (P= 0.029). Dorr classification was A or B in 24 patients in the modular group and 18 in the monobloc group (P = 0.006). Templating revealed that modular was not required in 25 patients.

Conclusions

As per our findings, it was determined that patients with a narrow femoral canal intraoperatively should not receive modular hemiarthroplasty. This is especially true for female patients with small femoral head and narrow femoral canal dimensions (Dorr A and B). They would require extensive careful planning. Surgical techniques should be explored through education intraoperatively to achieve lateralization during femoral stem preparation. This may avoid prolonged anesthetic time and achieve potential cost savings.

## Introduction

The National Hip Fracture Database (NHFD) reports that 66,313 patients presented with hip fractures in 2018 in the UK, with an estimated cost of £2 billion per year [1-4]. This is expected to rise with an aging population [4]. Hemiarthroplasty has been identified as the treatment of choice for elderly patients with displaced intracapsular femoral neck fracture, as outlined by the National Institute for Health and Clinical Excellence (NICE) [1-4]. NICE also recommended the use of a stem with a known track record [3,4]. Modular stems are sometimes favored over single-size monoblock hemiarthroplasty because of the different sizing options they offer to patients with a narrow femoral canal. However, the modular implant is more expensive [4], thus increasing the costs of both inventory and instrumentation sterilization. This has recently been highlighted by the NHFD as well as by Getting It Right First Time, as the implant cost alone amounts to £10.6 million per year.

Our unit uses the monoblock Exeter Trauma Stem (ETS®) (Stryker Corp.; Kalamazoo, MI, USA) femoral stem size 1.5 with an offset of 40 mm. However, when patients are suspected during broaching to have a narrow femoral canal, the standard monoblock ETS® prosthesis is converted to a modular V40® stem (Stryker) with a bipolar head. Modular hemiarthroplasty has been shown to provide no added clinical benefit in patient-reported outcome measures, with studies suggesting that it functions biomechanically as a monoblock, with the inner bearing losing its mobility, and that there is no difference in acetabular wear resulting in reoperation [5-9]. The use of monoblock versus modular prosthesis has long been debated. Recent trials have reported no differences in terms of health outcomes between another traditional design, Thompson™ Hemi Hip Stem (DePuy Synthes Inc., Warsaw, IN, USA) monoblock hemiarthroplasty, and a modern cemented hemiarthroplasty, as demonstrated in the WHiTE 3:HEMI randomized control trial and various systematic reviews [9].

In this study, we aimed to investigate the factors affecting surgeons’ choice of monopolar hemiarthroplasty in the form of modular hip hemiarthroplasty and to determine whether it was justified. We hypothesized that there was no difference between modular and monoblock hip hemiarthroplasty in relation to the size of the femoral canal. We believe femoral neck fractures in osteoporotic bone do not require smaller-sized stems and, therefore, do not require the use of modular stem for sizing reasons. We hypothesized that the variation in routine practice has implications for implant cost.

## Materials and methods

**Figure 1:**
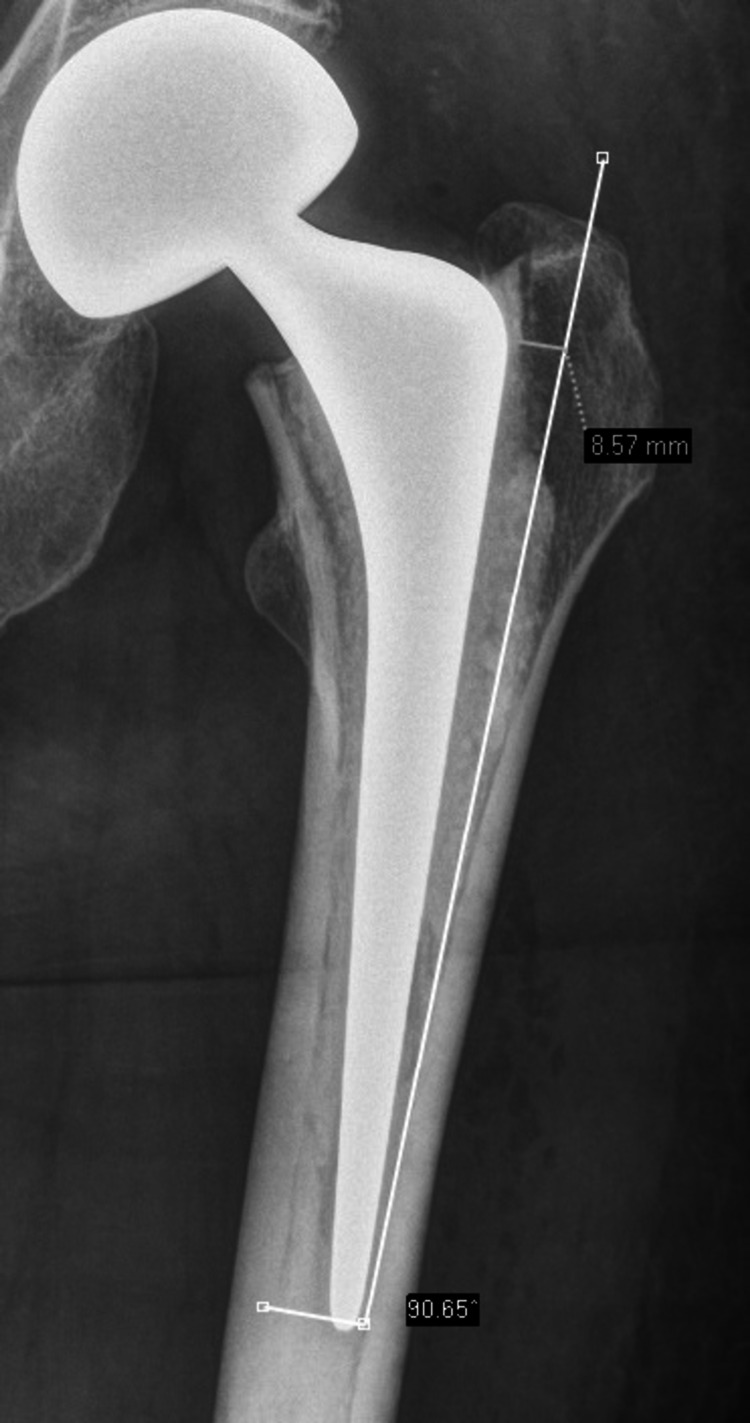
Example of measuring greater trochanter left (lateral wall) that was not appropriately taken off during stem preparation. A line is drawn parallel to the axis of the femur along the medial border of the lateral femoral shaft cortex, with lateral wall measured at the tip of the bipolar V40® stem.

We retrospectively reviewed all patients who sustained a femoral neck fracture and were admitted to our major trauma center from March 2013 to December 2016. We identified all patients who received bipolar hemiarthroplasty and monopolar Hemiarthroplasty and identified a patient-matched group selected at random who underwent monoblock hemiarthroplasty for the purpose of comparison. Of all the patients that received monobloc hemiarthroplasty, the patients were randomly chosen using a random number generator in that cohort and were found to have similar demographics to the comparative group hence the study design was a matched case-cohort study. We included patients who underwent modular hemiarthroplasty for the indication of a narrow femoral canal. We then retrospectively reviewed the operative notes on demographics, grade of surgeon, and femoral head size. We reviewed all radiographs and classified them using Dorr classification and thickness of the greater trochanter radiologically. On postoperative films, the thickness of the greater trochanter was defined as the bony prominence protruding beyond a line drawn at the medial border of the lateral femoral cortex, as illustrated in Figure 1.

**Figure 2:**
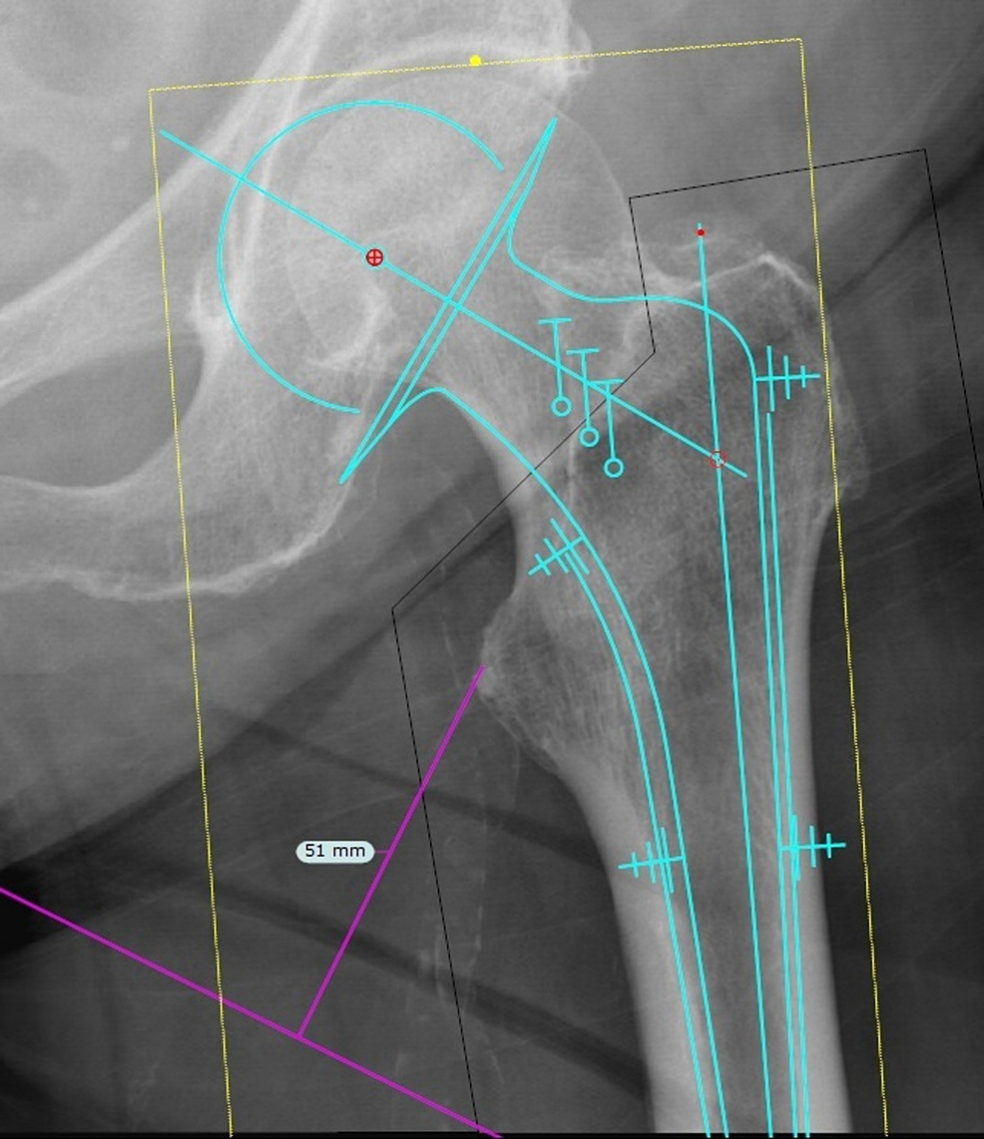
A patient who sustained a right femoral neck fracture was templated on TraumaCad using the ETS® stem, with evidence of prosthesis fitting the canal, but not achieving a 2-mm cement mantle.

Finally, we templated all modular hemiarthroplasty using TraumaCad software (Brainlab AG, Munich, Germany) with the Stryker ETS® template on the contralateral hip using the preoperative pelvic X-ray, as illustrated in Figure 2, in fitting an ETS® prosthesis incorporating a 2-mm cement mantle.

Statistical analysis 

Data were collected using the Medway patient administration and electronic patient record (PAS/EPR) system (System C Healthcare; Maidstone, Kent, UK) and radiographs reviewed on Kodak CARESTREAM picture archiving and communication system (Eastman Kodak, Inc./Carestream Health; Gland, Switzerland). Data analysis was performed using GraphPad Prism version 7 for Windows (GraphPad Software, San Diego, CA, USA). The Shapiro-Wilk test was used to determine normality, with parametric data analyzed using Student’s t-test and Fisher’s exact test. Differences with a p-value less than 0.05 were considered statistically significant. This study did not require ethical approval.

## Results

Of the 553 monoblock hemiarthroplasties (female, n = 354, 64.0%) and 30 modular hemiarthroplasties (female, n = 27, 100%) (P = 0.0007) performed between March 2013 and December 2016, 3 were excluded because they were performed for a different indication. After applying the inclusion criterion, which was the indication of a narrow femoral canal, 27 patients were included. This group was then studied in more detail and compared to a randomly matched patient group. The results are shown in Table 1.

**Table 1:** Summary of results between monoblock hemiarthroplasty and modular hemiarthroplasty for patients with femoral neck fracture. *Rounded up to the thousandth decimal place.

	Monoblock hemiarthroplasty (n = 27)	Modular hemiarthroplasty (n = 27)	P-value
Age	81.93 ± 7.5	80.22 ± 8.3	0.430
Head size (mm)	46.7 ± 3.6	44.07 ± 1.5	0.001*
Sex	17 females	27 females	0.0007*
Surgery performed by Consultant	7	8	1.000
Greater trochanter left (mm)	4.7 ± 3.9, n = 9 with unsatisfactory lateralization of greater trochanter	7 ± 2.9, n = 18 with unsatisfactory lateralization of greater trochanter	0.029*
Malaligned stem	4	14	0.008*
Dorr A	1	10	0.002*
Dorr B	17	14	0.003*
Dorr C	10	4	0.001*

The ratio of modular to monoblock hemiarthroplasty was 1:18 (or 5.6%). The average head sizes for the monoblock stem and modular stem were 46.7 mm ± 3.6 and 44.07 ± 1.5 (P = 0.001), respectively. There were four malaligned stems in the monoblock group versus 14 in the modular group (P = 0.008). In the monoblock group, 8 patients (4.7 mm ± 3.9) had unsatisfactory lateralization, compared with 18 (7 mm ± 2.9) in the modular group (P = 0.029). There were significantly more patients with Dorr A and B classification in the modular group (n = 24) than in the monoblock group (n = 18) (P = 0.006).

We further analyzed the modular hemiarthroplasty subgroup. We found that there were no significant differences (P = 0.237) between alignment of the femoral stem and lack of rasping on the lateral wall. We also templated all radiographs with TraumaCad to identify whether these patients were suitable for an ETS® implant, as illustrated in Figure 2. We found that all patients had sufficient femoral canal to accommodate an ETS® implant, with only two patients (7.4%) not achieving the minimum 2-mm cement mantle of an appropriately aligned insert.

## Discussion

Among the total hip arthroplasty patients, implant survival at 10 years did not differ between patients with a thin cement mantle and those with a standard technique with a 2-mm mantle [10]. The technique involves a thinner mantle, with the canal reamed to the same size as the prosthesis. Hence, we do not think that achieving a 2-mm cement mantle is crucial, particularly in this cohort of patients.

The retrospective radiologic templating we conducted gives insight into the misconception of requiring a smaller-sized stem in patients with femoral neck fracture. Although we do not advocate templating as a routine practice, our study demonstrates that Dorr A and B female patients are more likely to be mistakenly determined to require a smaller-sized femoral stem and converted to a modular stem.

In the UK, hemiarthroplasty for femoral neck fractures is commonly performed by junior surgeons in training under consultant supervision. Surgical technique in achieving good lateralization is often neglected, as illustrated by malaligned stems, often into varus (P = 0.008), as shown in Table 1. We, therefore, encourage the teaching of good surgical technique in femoral preparation, with sufficient lateralization, during young surgeons’ early years of training.

In 2017, 9.5% of all hip fractures (both cemented and uncemented) in which the indications were not reported were managed with modular, whereas 33.5% were managed with unipolar hemiarthroplasty, a ratio of 1:3.5. Despite similar clinical outcomes between monobloc and modular hemiarthroplasty reported in a recent meta-analysis and randomized control trials [4-9], with respect to monopolar or BH, the NICE guidance on hip fracture management does not favor one over the other [6]. The four-year surveillance 2015 - Hip fracture (2011) NICE guideline CG 124 [1] has, however, addressed the lower cost of monopolar prosthesis with no difference in outcomes between monopolar and modular. In the 2019 NHFD report, the cost difference between cemented hemiarthroplasty implants differed by means of £277 for the ETS® and £747 for the Exeter V40 stem + head + bipolar head), for a savings of £470.

Financial pressure on the NHS is increasing, and innovative service redesign projects are crucial to delivering current service at a lower cost. Using a modular implant will incur extra implant, inventory, and instrumentation sterilization costs. For our surgical department, the price of a monopolar prosthesis is £318.50; that of a modular implant (the bipolar head and Exeter stem) is £809.00, a difference of £571.50 for the prosthesis alone. The modular implant also incurs an additional £57.28 for sterilization of instrument trays. Over the period of the retrospective review, 27 modular implants, and an associated cost of £16,977, could have been avoided within our unit. We recognize that within the UK, practices can vary and indications for the modular implant differ, with some centers using it routinely. This information is currently unavailable in the NHFD2.

According to NHFD 2017 Annual Report, 65,645 patients were admitted with hip fracture, of whom 9.5 % (or 6236) underwent modular hip hemiarthroplasty. Because there are no clinical benefits of modular hip hemiarthroplasty in these patients and monoblock hemiarthroplasty could be implemented instead, this represents a potential cost savings of £2.9 million per year.

The limitations of this present study include its retrospective nature and the small sample size for analysis. In our institute solely monopolar modular hemiarthroplasties for comparison with the monobloc system. However, we were able to show that all patients had sufficient femoral canal size to accommodate an ETS® stem. Furthermore, Dorr classification was assigned using only AP pelvis views. Often, postoperative films were used when preoperative films were inadequate to measure the medullary canal at 10 cm, as suggested by Dorr et al. [11], because of the difficulty in standardizing films in this group of patients. This was compensated for by using any available AP pelvis films, both pre- and postoperatively, to achieve 10 cm. Lateral femur X-rays are not routinely taken within our unit because of the risk of aggravating the patient’s pain.

## Conclusions

Female patients with intracapsular femoral neck fracture with small femoral head size, Dorr A and B, require careful preoperative consideration and templating to avoid any unnecessary change of plan and incurring extra cost. We believe the learning curve for stem canal preparation for both trauma patients and patients undergoing hip arthroplasty is steep. Better surgical techniques should be explored through intraoperative education and departmental teaching to achieve adequate lateralization during femoral stem preparation. This can prevent unnecessarily long operative durations and achieve potential cost savings.
